# Medical News and Miscellany

**Published:** 1896-05

**Authors:** 


### Medical News and Miscellany.

**Dr. W. H. Seale** has removed from Marquez, Leon County, to Center Point, Kerr County.

**Dr. O. M. Conerly** has removed from Bivins to El Paso. Dr. Conerly will devote his time exclusively to diseases of the eye, ear and nose.

**Dr. T. J. Bennett,** of the *Texas Medical News*, attended the meeting of railroad surgeons at St. Louis last week. He is loca surgeon H. & T. C. R. R. at Austin.

**Dr. Sam R. Burroughs** will shortly remove from Raymond, Leon Connty, to Buffalo, same county, to get in touch with civilization, i. e., to get on a railroad.

**The copartnership** between Drs. Chilton & McReynolds, oculists, of Dallas, is dissolved. Dr. McReynolds has opened an office, and continues the practice of his specialty—*solus*.

**Dr. J. S. Carter,** of Dallas, a prominent physician and an old Confederate surgeon, formerly stationed at Jackson, Miss., died in Dallas about May 1st, after an illness of about a year.

**Dr. Wm. Caston,** a well known oculist of Corsicana and a member of the Texas State Medical Association, died at his home April 27th. Dr. Caston was a native of Hinds county, Mississippi.

**The American Medical Association** will meet next year at Philadelphia. At date of going to press no news of the meeting had been received, except that "Dr Nichols" had been elected president.

**A doctor** is needed at Lititia, a small station on the M., K. & T. R. R., twenty-five miles west of Houston; nearest doctor nine miles. Mr. F. V. Daniel, at Addicks P. O., Harris county, will give particulars if asked. A sober doctor wanted.

**Married.**—At Grace Church, Cuero, Texas, April 30th (ult.), Dr. Jos. H. Reuss to Miss Meta, daughter of Mr. and Mrs. Emil Reiffert, all of that city. The JOURNAL extends its congratulations and best wishes to the handsome young doctor and his fair bride.

**Send to Fort Worth Pharmacy Co.** for a catalogue of Surgical Instruments. With every order to the amount of $5, they will give a year's subscription to the *Red Back* as a premium, provided you are not already a subscriber. The JOURNAL endorses this house.

---

**Jenner Centennial.**—On May 22 (inst.), a Jenner Centennial will be held at Marietta, Pa., at the vaccine farms of Dr. H. M. Alexander & Co., and an invitation is extended to all physicians and their families to attend. An attractive programme has been prepared for the occasion.

---

**Hymeneal.**—The JOURNAL is in receipt of cards announcing the marriage of Prof. J. E. Thompson, of the Medical Department of the University of Texas, to Miss Eleanor Waters, to take place in Galveston on the 16th of May, inst. The JOURNAL extends congratulations and best wishes.

---

**Queer Place for a Sanitarium.**—Dr. W. Thornton Parker, in *Journal American Medical Association* for March 21, says: "I am much interested in a contribution by Dr. Bratton, concerning an arid-region Sanitarium in the New York *Medical Journal.*" What does the New York *Medical Journal* want with it? Is it for played-out editors whose brain has become an arid region? We have all heard of the man digging a well with a Roman nose! I read lately in a newspaper, "calculated to do an ordinary man with a family up."

---

**The Journal** regrets exceedingly to learn that Dr. A. P. Brown, of Fort Worth, ex-President of the Texas State Medical Association—a few days after adjournment of the meeting in that city—met with a painful and serious accident. His "Hambletonian" mare ran away, overturning the phaeton, fracturing upper and lower end of right fibula and dislocating the ankle. At this writing the doctor is resting easy, and is trying, he says, to exercise some of that patience and resignation which for sixty years has been preached to him.

---

**Antiphthisin.**—The New Orleans Faculty of Medicine have, through a commission of leading members, thoroughly tested the claims of antiphthisin, and make a rather favorable report. They say that upon the whole "antiphthisin does seem to have curative, and not simply palliative qualities." The remedy was tried on a number of cases of tuberculosis in Charity Hospital, with results quite gratifying, if not entirely satisfactory. The names of Professor Elliott, Souchon, Matas, Parham, Loeber, Jos. Holt, and other distinguished physicians, are appended to the report.

---

**In all the** Southwestern States, consisting of Texas, Louisiana, Mississippi, Arkansas, Oklahoma and the Indian Territory, the largest circulation credited to any publication devoted to medicine and surgery is accorded to the TEXAS MEDICAL JOURNAL, a monthly, published at Austin, Texas, and the publishers of the American Newspaper Directory will guarantee the accuracy of the circulation rating accorded to this paper by a reward of one hundred dollars, payable to the first person who successfully assails it.—*From Printers' Ink, issue of April 29th, 1896.*

---

**I. & G. N. Excursions.**—For the Christian Endeavor meeting, San Antonio, June 9–11; State Teachers' Association meeting, Austin, June 16–19, and B. Y. P. U., and State Sunday-School Convention, San Antonio, June 23–27, the I. & G. N. will make reduced rates to $5.00 maximum for round trip.

Excursions to Monterey and City of Mexico via Laredo will be run following each event.

Call on agent for full information.

D. J. PRICE, A. G. P. A.,
Palestine, Texas.

---

**We are** requested by the Fort Worth Pharmacy Company, of Fort Worth, Texas, to say, that they will be pleased, upon application, to mail their surgical instrument catalogue and price list to any member of the profession.

Their catalogue quotes over six thousand articles, and gives about the same number of cuts or figures of the articles.

They will also be pleased, at request, to mail special catalogues of electric batteries, microscopes, physician's chairs and tables and compressed air machines, which are now so successfully used by specialists in the treatment of throat and catarrhal diseases.

---

**A New Medical Society.**—Drs. Sharpe and Cummings, members of the 20th Judicial District Board of Medical Examiners, have issued a call for a meeting of physicians at Hearne on the 12th and 13th inst., to organize a District Medical Association, and announce a handsome program; a fine list of papers and a banquet constitute the principal features. The JOURNAL

acknowledges receipt of an invitation and the announcement and program, too late for insertion in this issue. This is a commendable move which we hope to see very generally followed by other district boards. We must thoroughly organize the profession.

---

**Political Conventions.**—For the following political conventions the Santa Fe will make round-trip rates of one fare from all of its Texas and Indian Territory points:

National Prohibition Convention, Pittsburg, Pa., May 27th, 1896.

National Republican Convention, St. Louis, Mo., June 16th, 1896.

National People's Convention, St. Louis, Mo., July 22nd, 1896.

For particulars as to limits and time cards, call upon any Santa Fe agent or write to

W. S. Keenan,
General Passenger Agent, Galveston.

---

**Texas State Medical Association.**—Change in Terms and Plan of Admission:—Notice is hereby given to members of every county and district medical society in Texas in affiliation with the State organization that they can now become members of the State Association, by sending to the treasurer, Dr. J. Larendon, Houston, a certificate of membership, duly signed by the president, accompanied by $5.00 annual dues. The initiation fee has been dispensed with. The payment above mentioned entitles the applicant to the Transactions. Blank forms of application will be furnished to the various local officers as soon as possible; in the mean time a certificate in writing will answer the purpose. It is necessary that application should be made as speedily as possible, in order that the Publishing Committee may know how many volumes of the Transactions to contract for. Every secretary of local societies in the State is earnestly requested to furnish me with a list of officers and members, and to do so at an early date.

H. A. West, Sec'y T. S. M. A.

Galveston, May 11, 1896.

---

**Commencement Exercises** of Texas Medical College (Medical Department to Univerity of Texas, Galveston) will be held at Grand Opera House, Friday, May 15th. Engraved ornamental invitations have been issued. There are thirty-six graduates in medicine and nine in pharmacy—45, or about 20 per cent of the matriculants; class inclusive 236. The graduates are as follows:

L. Anderson, W. E. Ashton, P. D. Barnhill, H. A. Barr, W. H. Blackburn, J. M. Campbell, M. B. Canon, W. S. Carruthers, W. J. Crook, F. G. Daehne, W. T. Davidson, G. E. Delaney, F. B. Eldman, B. W. Fontaine, B. Frenkel, F. C. Fuller, C. B. Garner, D. D. Hamilton, F. B. Hogg, F. R. Karbach, N. Long, W. E. Luter, A. F. Lumpkin, W. Mercer, H. P. Moor, J. T. Moore, R. L. McMahon, E. B. Osborne, J. E. Prindgen, G. P. Rains, J. C. Ralston, J. L. Short, G. M. Smith, Jr., W. R. Smith, E. A. Watters, J. E. Wilson and W. P. Woodall.

The members of the pharmacy class are as follows: C. W. Sparks, Carol Clark, J. E. Fuller, H. Koester, C. E. Koerth, T. J. Morris, W. Maymon, J. R. Hodges and H. J. Warner.

---

**Died.**—At the home of her parents, in Cleburne, Tex., at 8 a. m., Thursday, May 7th inst., after a lingering illness, Miss Hattie Lou Osborn, daughter of Dr. J. D. Osborn, and granddaughter of Dr. T. C. Osborn.

The Journal is greatly pained at the announcement of the death of this most estimable and accomplished young lady. Gifted by nature—she had by study added many accomplishments—which like jewels adorned her naturally graceful and charming character. As an elocutionist she possessed rare powers, and many of the Journal's readers may recall her splendid contest at the capital some time ago for the State medal. Miss Osborn was a graduate and a favorite pupil of Belmont College, Nashville. She had a host of attached friends throughout the South devoted to her beause of her lovely, rare, unselfish nature, who will share with her grief-stricken parents the great grief of her so untimely death. All loved her who ever came within the charm of her presence. The Journal extends its condolence to the bereaved family.

---

### CIRCULAR TO PHYSICIANS.

### Eye and Ear Charity Hospital.

Through the co-operation of some of the good women of Texas, there has been founded in the city of Austin an Eye and Ear Charity Hospital for patients who are unable to pay for board and treatment. Arrangements have also been made to receive and treat patients who are able to pay. Only trained nurses are employed, and the surgeon in charge is daily assisted by Dr. Jos. S. Wooten.

I take this method of announcing to the profession that the hospital was opened September 1st, 1895, and has been in operation since that date. Your co-operation is respectfully solicited, and a cordial invitation is extended to you, when in the city, to visit the hospital and see for yourself that it is properly conducted.

For further information addresss

H. L. Hilgartner, M. D.
Surgeon in Charge.

